# Altered gut microbiome profile in patients with knee osteoarthritis

**DOI:** 10.3389/fmicb.2023.1153424

**Published:** 2023-05-12

**Authors:** Xi Wang, Yifan Wu, Yanli Liu, Feihong Chen, Sijie Chen, Feiyu Zhang, Shujin Li, Chaowei Wang, Yi Gong, Ruitian Huang, Minhan Hu, Yujie Ning, Hongmou Zhao, Xiong Guo

**Affiliations:** ^1^Department of Occupational and Environmental Health, School of Public Health, Xi'an Jiaotong University Health Science Center, Xi’an, Shaanxi, China; ^2^Key Laboratory of Trace Elements and Endemic Diseases, School of Public Health, Xi’an Jiaotong University Health Science Center, National Health and Family Planning Commission, Xi’an, Shaanxi, China; ^3^Foot and Ankle Surgery Department, Honghui Hospital of Xi’an Jiaotong University, Xi’an, Shaanxi, China; ^4^Clinical Research Center for Endemic Disease of Shaanxi Province, The Second Affiliated Hospital of Xi'an Jiaotong University, Xi'an, Shaanxi, China

**Keywords:** osteoarthritis, 16s rDNA sequencing, metagenomic sequencing, cartilage-gut-microbiome axis, gut microbiota biomarkers

## Abstract

**Introduction:**

Osteoarthritis (OA) is a kind of chronic, degenerative disorder with unknown causes. In this study, we aimed to improve our understanding of the gut microbiota profile in patients with knee OA.

**Methods:**

16S rDNA gene sequencing was performed to detect the gut microbiota in fecal samples collected from the patients with OA (*n* = 32) and normal control (NC, *n* = 57). Then the metagenomic sequencing was used to identify the genes or functions linked with gut microbial changes at the species level in the fecal samples from patients with OA and NC groups.

**Results:**

The Proteobacteria was identified as dominant bacteria in OA group. We identified 81 genera resulted significantly different in abundance between OA and NC. The abundance of *Agathobacter*, *Ruminococcus*, *Roseburia*, *Subdoligranulum*, and *Lactobacillus* showed significant decrease in the OA compared to the NC. The abundance of genera *Prevotella_7*, *Clostridium*, *Flavonifractor* and *Klebsiella* were increasing in the OA group, and the families *Lactobacillaceae*, *Christensenellaceae*, *Clostridiaceae_1* and *Acidaminococcaceae* were increasing in the NC. The metagenomic sequencing showed that the abundance of *Bacteroides stercoris*, *Bacteroides vulgatus* and *Bacteroides uniformis* at the species level were significantly decreasing in the OA, and the abundance of *Escherichia coli*, *Klebsiella pneumoniae*, *Shigella flexneri* and *Streptococcus salivarius* were significantly increased in OA.

**Discussion:**

The results of our study interpret a comprehensive profile of the gut microbiota in patients with knee OA and offer the evidence that the cartilage-gut-microbiome axis could play a crucial role in underlying the mechanisms and pathogenesis of OA.

## Introduction

Osteoarthritis (OA) is a kind of chronic, degenerative disorder with unknown causes. The most widespread osteochondrosis causes the most common pathological changes, including cartilage degradation, abnormal bone formation, and synovial membrane inflammation (Liu et al., 2019). The clinical manifestations of OA include severe pain in joints, limitation of movement and malformation of various joints (hands, knees and ankles). OA was identified as a disorder of comprehensive causes, such as sex, aging, athletic injury and being overweight (Johnson and Hunter, 2014). Knee OA mainly affects the articular cartilage of elderly, individuals and always progresses to severe disability due to the unknown pathogenesis and lack of effective treatments. Therefore, research on the biological mechanism and cartilage degeneration processes is crucial to determine pathogenesis and develop treatments for OA. The microbiota is the complex and dynamic microbial ecosystem living within the intestine, including many organic compounds and their metabolites (Hernandez et al., 2016; Lloyd-Price et al., 2016; Kundu et al., 2017). Maintaining the equilibrium of the microbial ecology in the human digestive tract by harboring 10^14 resident microorganisms and more than 1,000 bacterial species is one of the most crucial functions of the microbiota (Sommer and Backhed, 2013).

For the past few years, there has been a growing understanding of a potential gut-cartilage-microbiota axis involved in the occurrence and development of joint diseases, such as OA. Many studies have suggested that dysbiosis of the gut microbiota is related to animal models of OA and humans (Berthelot et al., 2019; Szychlinska et al., 2019; Favazzo et al., 2020). Previous studies revealed that circulating inflammatory molecules such as lipopolysaccharide (LPS) were correlated with OA severity in animal models and in OA patients, which suggested that proinflammatory metabolites derived from the microbiome might play a crucial role in the development of OA.

Therefore, 16S rDNA gene sequencing and metagenomic sequencing were performed to investigate the alterations in the microbiome of OA. The results of our study provide a comprehensive profile of the gut microbiota in patients with knee OA and offer evidence that the cartilage-gut-microbiome axis could play a crucial role in underlying the mechanisms and pathogenesis of OA.

## Materials and methods

### Research design and sample recruitment

In this study, 32 patients with knee OA from Xi’an Hong Hui Hospital in Shaanxi province were recruited and were diagnosed strictly by the criteria for diagnosis of arthritis (Modified Outerbridge Classification). In addition, 57 normal controls (NCs) were recruited from Shaanxi province. To exclude the differences caused by dietary, the individuals with comparable eating habits were selected into this study. For all OA and NC subjects, the exclusion criteria was as follow: (1) Suffering or suffered from some other arthritic diseases (such as rheumatoid arthritis, gout, or skeletal fluorosis), (2) Any other type of chronic disease, for example diabetes, coronary heart disease and hypertension, (3) Accepted any remedies in the past 180 days, (4) A history of sick with irritable bowel syndrome (IBS), inflammatory bowel disease (IBD) or complications of complete intestinal obstruction, and (5) Intake of antibiotics, probiotics, prebiotics, or synbiotics during past 60 days. The general clinical data of all subjects were included age, sex, educational background, and body mass index (BMI). All patients with OA and NC were Shaanxi Han Chinese with the same geographic areas and similar habits of diet. All qualified feces samples were in triplicate per sample, packed into three freezer tubes, and frozen in liquid nitrogen overnight and preserved at −80°C for further research. Clinical information was collected from patient records. All donors signed a written informed consent form. All subjects were of Chinese Han lineage. The study protocol was approved by the ethics committee of Xi’an Jiaotong University (Approval No. 2022–685). The study’s Chinese Clinical Trails Registry number is ChiCTR2300070898.

### Stool DNA extractions

DNA from feces samples of OA and NC groups were extracted using The E.Z.N.A.^®^ Stool DNA Kit (D4015-02, Omega, Inc., United States) according to manufacturer’s instructions. The reagent which was designed to uncover DNA from trace amounts of sample has been shown to be effective for the preparation of DNA of most bacteria. Sample blanks consisted of unused swabs processed through DNA extraction and tested to contain no DNA amplicons. The total DNA was eluted in 50 μL of Elution buffer by a modification of the procedure described by manufacturer (Omega) and stored at −80°C until measurement in the PCR by LC-BIO TECHNOLOGIES (HANGZHOU) CO., LTD., Hang Zhou, Zhejiang Province, China.

### 16S rDNA gene and metagenomic sequencing

Primers 341F (5’-CCTACGGGNGGCWGCAG-3′) and 805R (5’-GACTACHVGGTATCTAATCC -3′; Logue et al., 2016) were used to amplify the V3-V4 region of the small-subunit (16S) rRNA gene in prokaryotes (bacteria and archaea). The final concentration of primers can be found in Supplementary materials. The 5′ ends of the primers were labeled with a specific barcode for each sample, and the universal primers were sequenced. After the preparation of a total volume of 25 μL reaction mixture including 25 ng of template DNA, 12.5 μL PCR Premix, 2.5 μL of each primer, and PCR-grade water which used to adjust the volume, we performed PCR amplification. PCR conditions for amplication of the prokaryotic 16S fragments include: initial denaturation at 98°C for 30 s; 32 cycles of denaturation at 98°C for 10 s, annealing at 54°C for 30 s, and extension at 72°C for 45 s; followed by a final extension at 72°C for 10 min. The PCR products were confirmed with 2% agarose gel electrophoresis. Throughout the DNA extraction process, ultrapure water, instead of sample solution, was used to exclude the possibility of false-positive PCR results as a negative control. The PCR products were purified by AMPure XT beads (Beckman Coulter Genomics, Danvers, MA, United States) and quantified by Qubit (Invitrogen, United States). The amplicon pools were prepared for sequencing and the size and quantity of the amplicon library were assessed on an Agilent 2100 Bioanalyzer (Agilent, United States) and with the Library Quantification Kit for Illumina (Kapa Biosciences, Woburn, MA, United States), respectively. The libraries sequencing was performed on NovaSeq PE250 platform.

According to the manufacturer’s recommendations provided by LC-Bio, samples were sequenced on an Illumina NovaSeq platform. Based on unique barcode of each sample, paired-end reads were assigned and the truncation of the barcode and primer sequence were performed. Then paired-end reads were merged using FLASH. According to the fqtrim (v0.94), raw reads were quality-filtered under specific filtering conditions to obtain high quality clean tags. In addition, chimeric sequences were filtered using Vsearch software (v2.3.4). The feature table and feature sequence were acquired by (v1.26.0; Benjamin et al., 2016) and the bray-curtis was performed for distance method for PCoA and LEfSe. We investigated the Alpha diversity and beta diversity by QIIME2 (Evan et al., 2019), and completed the work of bacteria taxonomy using the relative abundance. The QIIME2 process and R (v3.5.2) were used to calculated Alpha and Beta diversity and draw the pictures, respectively. The sequence alignment of species annotation was performed by BLAST, with the SILVA and NT-16S alignment database. LEfSe and random forest model were performed as follow steps, first, detect all characteristic species using Kruskal Wallis rank sum test, and obtain significantly different species by detecting species abundance differences between different groups, second, to detect whether all subspecies of the significantly different species obtained in the previous step converge to the same classification level by Wilcoxon rank sum test, third, using linear discriminant analysis (LDA) to obtain the final differential species. Random forest is an efficient and learning algorithm based on decision trees, belonging to a nonlinear classifier.

According to the results of 16S sequencing, we selected 32 OA and 35 NC were performed metagenome sequencing. DNA library was constructed by TruSeq Nano DNA LT Library Preparation Kit (FC-121-4001). DNA was incubated with dsDNA Fragmentase(NEB, M0348S) for 30 min at 37°C to achieve DNA fragmentation. Library construction starts with fragmented DNA. By using a combination of fill-in reactions and exonuclease, activity to generate blunt-end DNA fragments, and size selection was performed with provided sample purification beads. An A-base was then added to the blunt ends of each strand, to connect them to the indexed adapters. The T base overhang was used to attach the adapter to the A-tail fragment DNA, which is present on every adapter. The full complement of sequencing primer hybridization sites was included in these adapters, for single, paired-end, and indexed reads. The final concentration of primers can be found in Supplementary materials. Attach single- or dual-index adapters with the fragments, and then apply PCR to amplify the ligated products according to the following conditions: initial denaturation at 95°C for 3 min; 8 cycles of denaturation at 98°C for 15 s, annealing at 60°Cfor 15 s, and extension at 72°C for 30 s; and then final extension at 72°C for 5 min.

In order to obtain valid reads for further analysis, the original sequencing reads were processed. Firstly, cutadapt (v1.9; Kechin et al., 2017) was used to remove the sequencing adapter from the sequencing reads. Secondly, fqtrim (v0.94) used a sliding-window algorithm to trim low quality reads. Thirdly, the use of bowtie2 (v2.2.0; Langmead and Salzberg, 2012; Langmead et al., 2018) aligned the reads and the host genome in order to remove host contamination. After obtaining high quality-filtered reads, they were *de novo* assembled by IDBA-UD (v1.1.1; Peng et al., 2012) to construct the metagenome for each sample. MetaGeneMark (v3.26; Zhu et al., 2010) predicted all coding regions (CDS) of metagenomic contigs. CD-HIT (v4.6.1; Li and Godzik, 2006) clustered CDS sequences of all samples to obtain unigenes. TPM was used to estimate the unigene abundance for a certain sample based on the number of aligned reads by bowtie2 (v2.2.0). Unigenes were compared with the NCBI NR database by DIAMOND v 0.9.14 to obtain the lowest common ancestor taxonomy of unigenes. Similarly, the functional annotation (GO, KEGG) of unigenes were obtained (Minoru and Susumu, 2000; Minoru et al., 2014). Fisher’s exact test was used to perform differential analysis at each taxonomic or functional or gene-wise level, which was based on the taxonomic and functional annotation of unigenes, as well as the abundance profile of unigenes.

### Statistical analyses

Pearson’s Chi-square test or Fisher’s exact test were performed to identified the significant differences in clinical characteristics. *p* < 0.05 means the differences were significant. GraphPad Prism 6 software (GraphPad software, Inc., San Diego, California, United States), R version 3.5.2 (R Foundation for Statistical Computing, Vienna, Austria) and Microsoft Excel (Microsoft Corporation, Seattle, WA, United States) were used to analyze the data in this manuscript.

## Results

### Clinical features of the subjects

The participants in this research were Han Chinese and had lived in Shaanxi Province for a significant amount of time. The mean age of patients with OA was 68.2 ± 3.5 years, and the mean age of NCs was 62 ± 2.4 years. No significant differences were found in age, sex ratio or BMI between the OA and NC groups (Table 1).

**Table 1:** Characteristics of participants in this study.

Characteristic	NC	OA	*p* value
Subjects (*n*)	57	32	
Male/Female	24/23	9/23	>0.05
Age (mean), years	62	67	>0.05
Degree	-	III^*^ (9)	
	-	IV^*^ (23)	
BMI	23.79	24.24	>0.05
TKA	-	Left	
ESR (mean), (mm/h)	9.3	13.8	>0.05
CRP (mean), (mg/dL)	0.17	0.24	>0.05
WBC (mean), (10^9^/L)	4.88	5.76	>0.05
Lymphocyte (mean), (10^9^/L)	0.26	0.37	>0.05
Monocyte (mean), (10^9^/L)	0.06	0.096	>0.05
BG (mean), (mmol/L)	5.28	5.06	>0.05
ALT (mean), (U/L)	14.7	16.3	>0.05
AST (mean), (U/L)	15.8	17.2	>0.05

^*^Grade III and IV OA patients according to the Kellgren Lawrence scoring system. TKA, total knee arthroplasty; ESR, erythrocyte sedimentation rate; CRP, C-reactive protein; WBC, white blood cell; BG, Blood glucose; ALT, glutamic-pyruvic transaminase; AST, aspartate transaminase.

### Alterations in gut microbiota composition in patients with OA and NCs based on 16S rDNA analysis

In present microbiota analysis, 4,832,198 high-quality 16S rDNA reads, with a median of 54,799 reads (range: 35,830 to 71,386) were acquired per sample (Supplementary Table S1). A total of 8,607 characteristics were obtained by sequencing 89 samples (Supplementary Table S2). Comprehensive details regarding the 16S rDNA data of all samples are presented (Supplementary Table S3).

**Figure 1:**
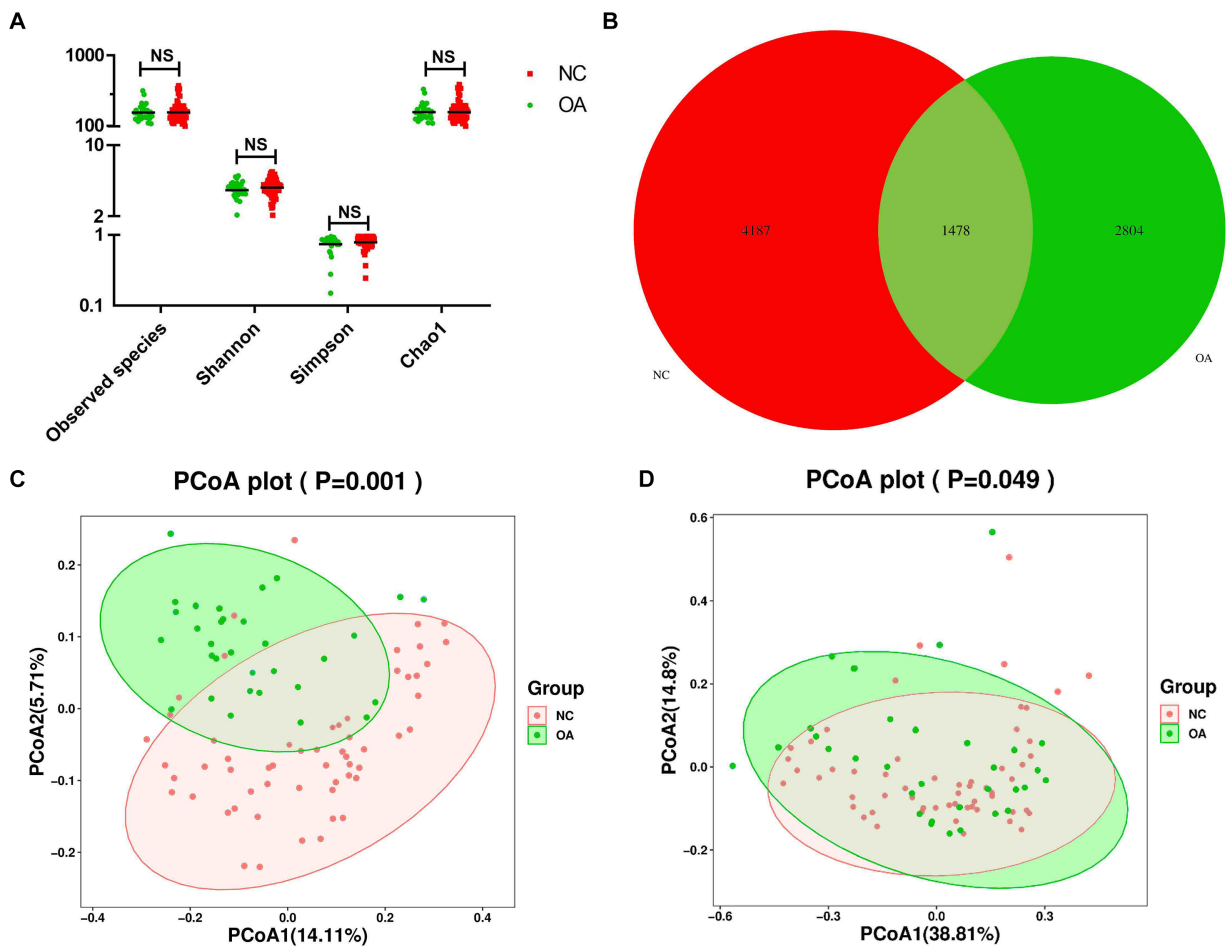
Gut microbiome diversity and structure analysis based on 16S rDNA sequencing data. **(A)** Species diversity differences in the OA and NC groups were estimated by the observed species, Shannon, Simpson, and Chao1 indices. NS, not significant. OA, patient in osteoarthritis group; and NC, normal control. **(B)** Venn diagram of the observed features in KBD and NC. **(C,D)** Principal coordinate analysis (PCoA) of the microbiota based on the unweighted (*p*= 0.001) and weighted (*p*= 0.049) UniFrac distance matrices for the OA and NC groups.

Alpha diversity and beta diversity between OA patients and normal controls were compared to assess the characteristics of the gut microbiome related to OA. According to the analysis results, the Shannon, observed species, and Chao1 indices did not show any statistically significant differences (Figure 1A; Supplementary Table S4). According to the Venn diagram, the OA group had 2,804 unique features, compared to 4,187 unique features in the normal control group. The two groups had a combined total of 1,478 features (Figure 1B). Using principal coordinates analysis (PCoA) based on unweighted and weighted UniFrac distance metrics, we found that the microbiome of the OA group was distinct from that of the NC group. We further performed an analysis of similarities (ANOSIM), and the results indicated that the structure of the gut microbiome of the OA group was significantly different from that of the NC group (ANOSIM, *r* = 0.25, *p* < 0.001, unweighted UniFrac, Figures 1C,D).

### Alterations in the composition of the fecal microflora associated with OA

**Figure 2:**
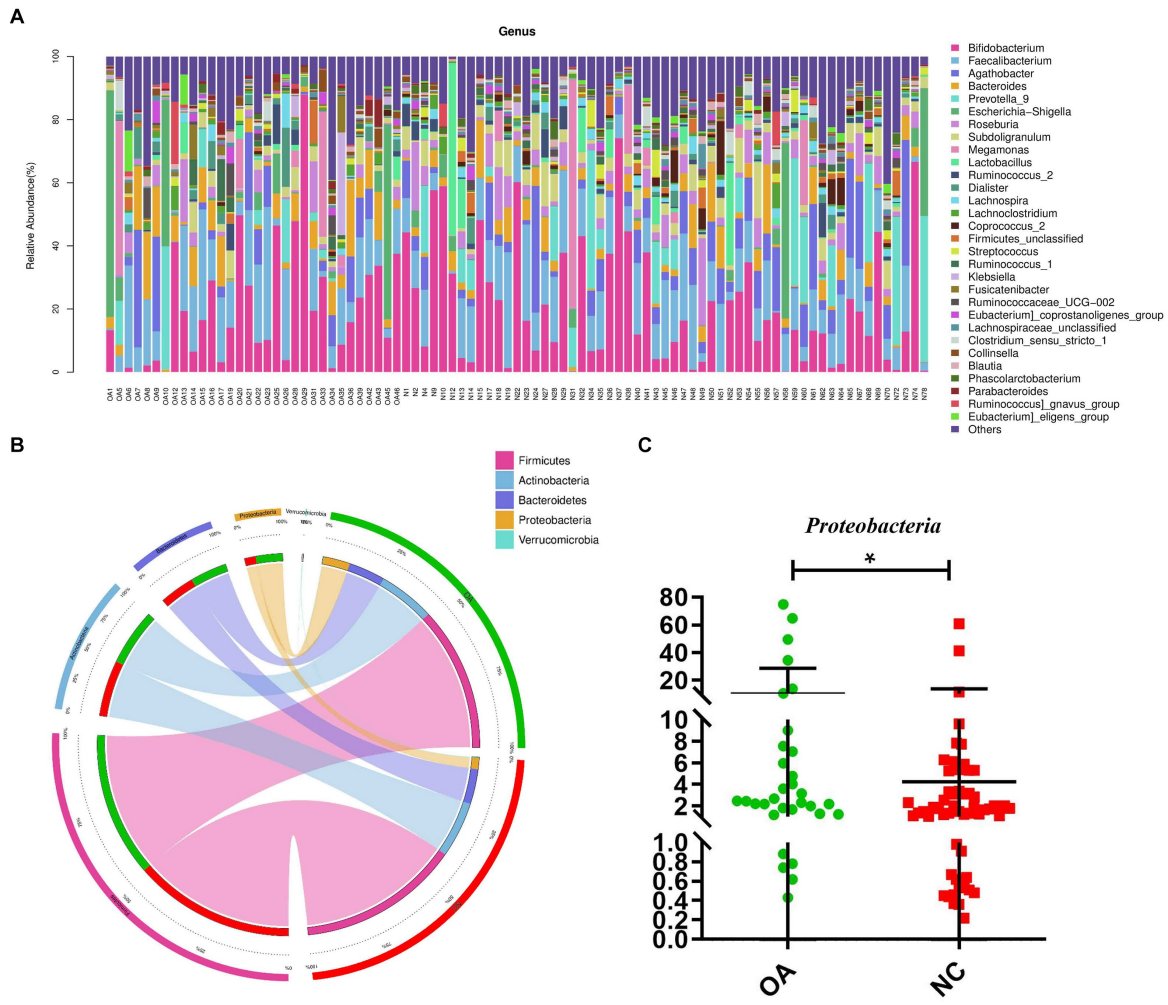
Gut microbiome differential analysis. **(A)** Component proportions of bacterial genus in each group; *n*= 32 for the OA group and *n*= 57 for the NC group. **(B)** Circos plot. The left side of the circle represents species and the right side represents sample groups, different colors represent different taxonomic categories and sample groups. From left to right, the thickness of the same color line in the inner ring represents the relative abundance of the species in different sample groups, from right to left, the thickness of the same color line in the inner ring represents the proportion of different species in the sample group. **(C)** The differences in abundance of *Proteobacteria* between the OA and NC group.

To evaluate the relative proportions of dominant taxa at the genus level in the OA and NC groups, microbial taxon assignment was carried out. The gut microbiota showed significant variations across the samples in each group (Figure 2A). The most dominant phylum was *Firmicutes*, with proportions of 54.53 and 60.99% of the characteristics in the OA and NC groups, respectively. Furthermore, the OA group had higher concentrations of *Actinobacteria* (21.37% versus 21.17%) and *Bacteroidetes* (13.84% versus 13.22%) than the NC group (Figure 2B; Supplementary Table S5).

At the phylum level, higher levels of *Proteobacteria* were characteristic of the OA group (Figure 2C). At the genus level, 81 distinct genera had significantly differing abundances across the two groups (Supplementary Table S6). Compared to the NC group, the abundances of *Agathobacter*, *Ruminococcus*, *Roseburia*, *Subdoligranulum* and *Lactobacillus* were shown to be considerably lower in the OA group among these discriminatory taxa (Supplementary Figure S1).

**Figure 3:**
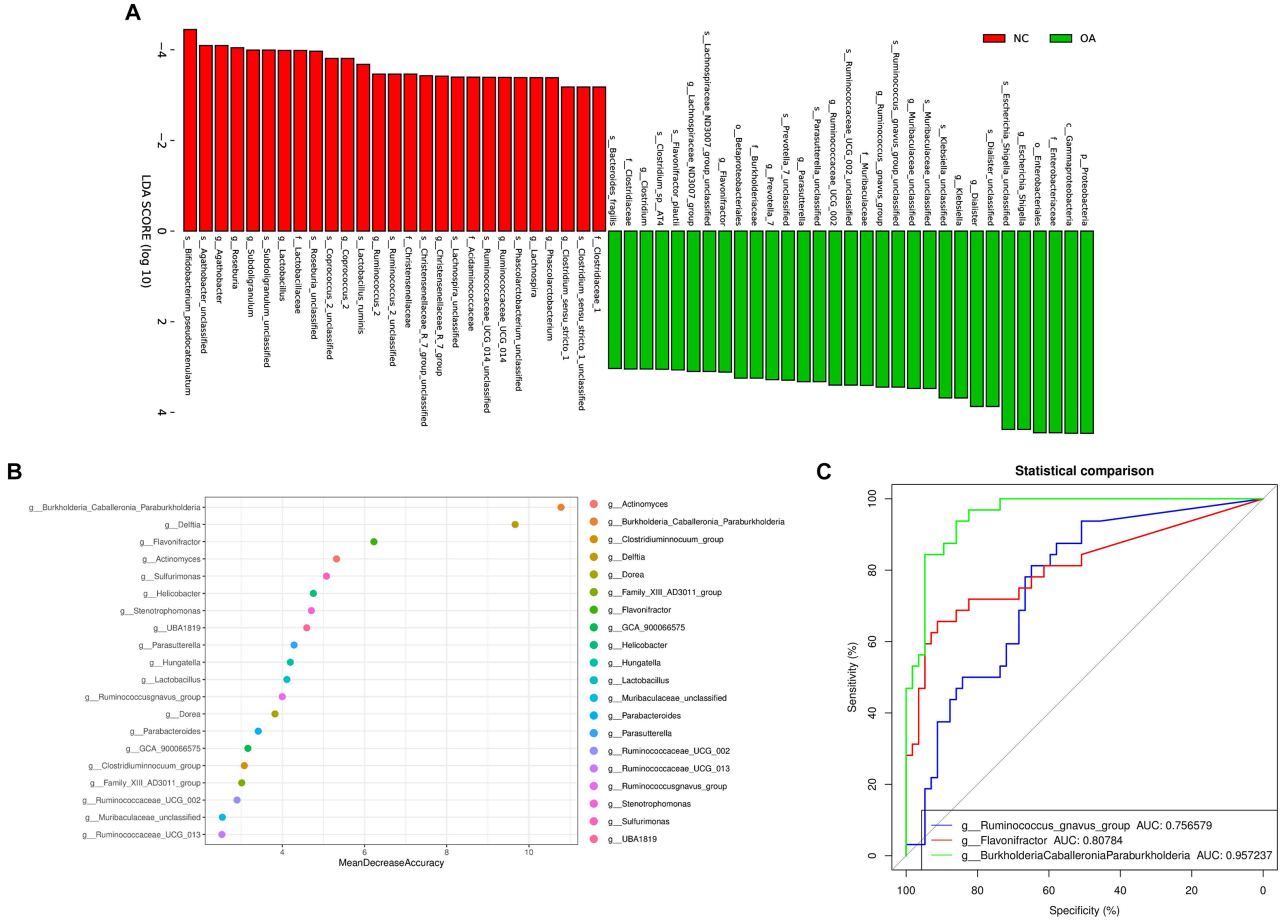
Gut microbiome differential and functional analysis. **(A)** Linear discriminant analysis (LDA) integrated with effect size (LEfSe). The differences in abundance between the OA and NC group. **(B)** Classification performance of a random forest model using 16 s rRNA genus abundance assessed by R random Forest package. **(C)** ROC curve displaying the top 3 biomarkers for classification between OA and NC. AUC, area under curve.

We found the specific bacteria linked to OA by combining linear discriminant analysis (LDA) and effect size (LEfSe) to generate a cladogram (Figure S2). *Proteobacteria* and *Escherichia_Shigella* levels increased in the OA group according to LDA distribution diagram analysis (LDA score > 3), indicating a significant alteration in the microbiota (Figure 3A). However, the *Coprococcus_2* levels of the OA group dramatically decreased (Figure 3B). In the NC group, the genera *Lactobacillaceae*, *Christensenellaceae*, *Clostridiaceae_1* and *Acidaminococcaceae* were more prevalent, but in the OA group, *Prevotella_7*, *Clostridium*, *Flavonifractor* and *Klebsiella* were more abundant (Supplementary Figure S2).

### The potential role of gut microbiota biomarkers in the risk assessment of OA

We discovered numerous possible diagnostic biomarkers that may be utilized to differentiate the OA and NC groups using a random forest model based on the differentially abundant species. The best discriminatory power was supplied by the optimal model, which used 20 genera (Figure 3B). The abovementioned analysis revealed substantial differences in the microbial community distribution between the OA and NC groups. Moreover, we established receiver operating characteristic (ROC) curves and calculated the area under the curve (AUC) values to investigate the potential utility of the identified bacterial biomarkers for discrimination of the OA and NC groups. The top 3 AUC values were for *BurkholderiaCaballeroniaParaburkholderia* with 95.72%, *Flavonifractor* with 80.78%, and *Ruminococcus_gnavus_group* with 75.65% (Figure 3C).

### Prediction of gene function in the gut microbiota

In this study, the gut microbial gene functions across the Clusters of Orthologous Genes (COGs), enzyme nomenclature (EC), Kyoto Encyclopedia of Genes and Genomes (KEGG), KEGG orthology functional orthologs (KO), protein families (PFAM) and protein families featuring curated multiple sequence alignments (TIGRFAM) databases between the OA and NC groups were analyzed using the phylogenetic investigation of communities by the method of reconstruction of unobserved states (PICRUSt2). We identified various significant functions, including glycogen biosynthesis and phosphatidylglycerol biosynthesis across the KEGG pathways (Supplementary Figure S3) and selenophosphate synthetase-related protein and phospholipase C in the COG database. Supplementary Figure S4 lists the top 30 identifiable functions of the above database.

### Alterations in gut microbiota composition in patients with OA and NCs based on metagenomic sequencing analysis

**Figure 4:**
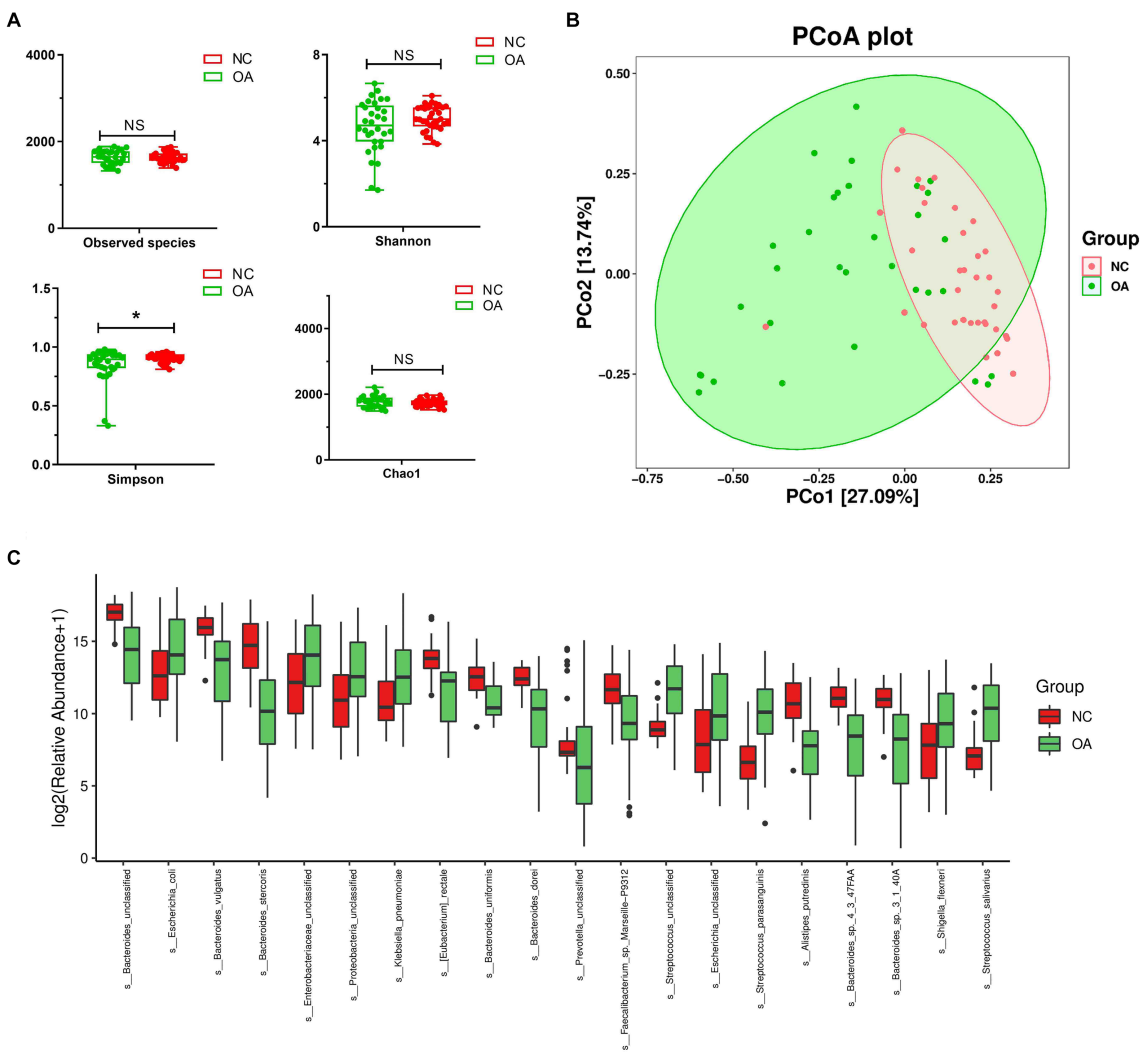
The gut microbiota differences in OA and NC group based on the metagenomic sequencing data. **(A)** Alpha diversity differences between the OA and NC groups were estimated by the observed species, Shannon, Simpson, and Chao1 indices. ^*^p < 0.05. OA, patients with OA group; NC, normal control group. **(B)** The PCoA analysis based on the Bray-curtis distance matrix between the OA and NC group at the species level (*p* = 0.001). **(C)** The relative abundance of top 20 species enriched in OA versus KBD. The box represents the interquartile ranges, inner line denotes the median; *n* = 32 for the OA group and *n* = 35 for the NC group based on the metagenomic sequencing data.

Stool samples collected from 32 OA patients and 35 NC subjects were used for metagenomic sequencing. According to the analysis, 149,563 genes were predicted (Supplementary Figures S5a–c). When compared to the OA samples, the NC group samples featured 6,527 unique genes (Supplementary Figure S5d). A total of 64,308 differentially expressed unigenes (including 48,572 upregulated and 15,736 downregulated unigenes) were found in the OA group compared to the NC group (Supplementary Figure S5e). Simpson indices revealed that the alpha diversity of the OA group was much lower than that of the NC group (Figure 4A). Using PCoA based on the Bray-Curtis distance matrix, it was possible to detect substantial variations in microbial composition between the OA and NC groups at the species level (Figure 4B). According to the results (Supplementary Table S8) *Bacteroides vulgatus*, *Bacteroides stercoris* and *Bacteroides uniformis* had lower abundances in the OA group than in the NC group (Figure 4C; Supplementary Figure S8), and the species *Escherichia coli*, *Klebsiella pneumoniae*, *Shigella flexneri* and *Streptococcus salivarius* had significantly higher abundances in the OA group than in the NC group (Figure 4C; Supplementary Table S7).

### Functional metagenomic analysis between the OA and NC groups

**Figure 5:**
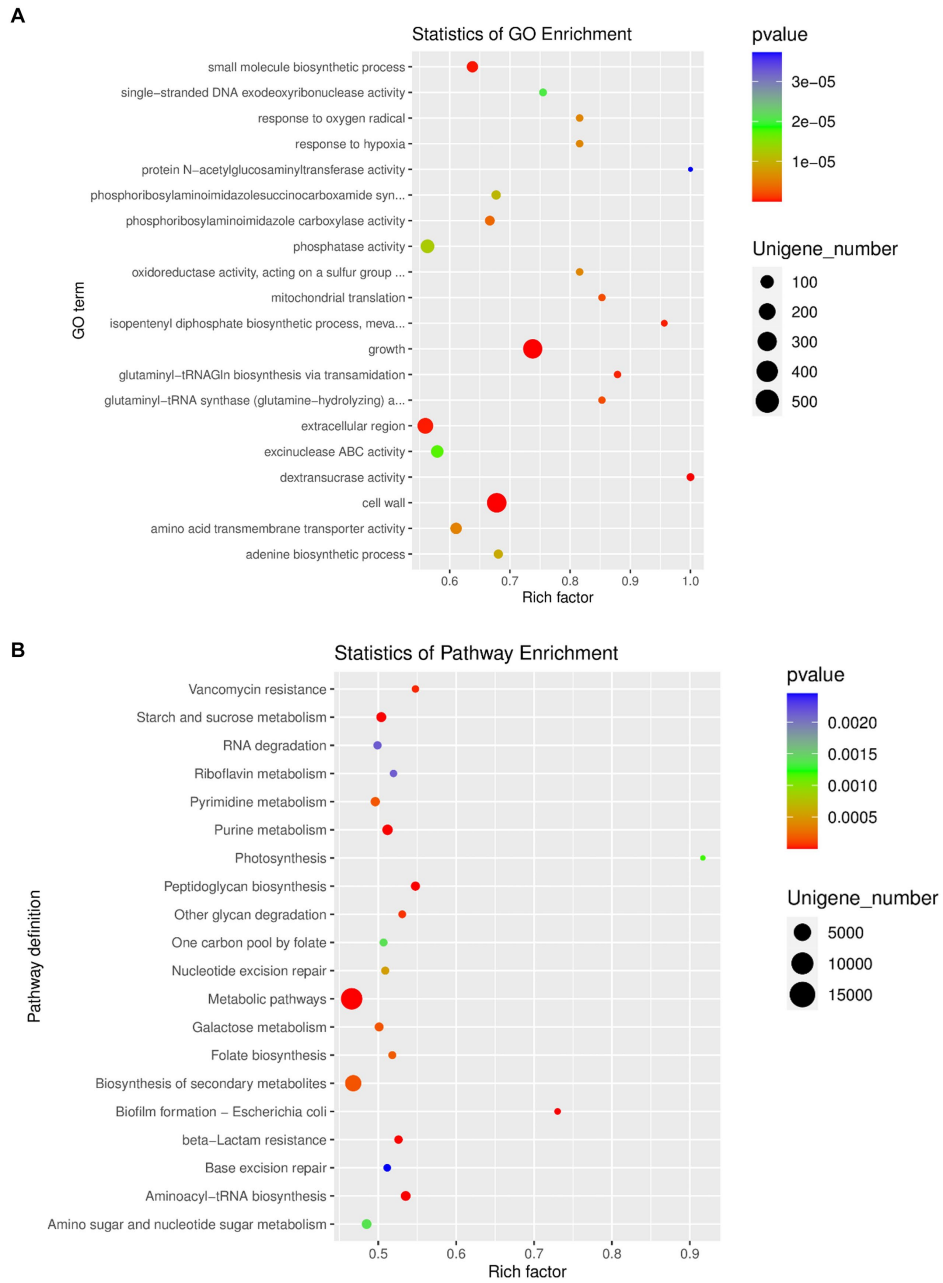
**(A)** GO enrichment analysis of differentially expressed unigenes between OA and NC group. **(B)** Pathway classification based on KEGG enrichment analysis of differentially expressed unigenes between OA and NC group.

The top 10 GO items of the three types of definitions among the GO database were chosen to perform the GO function classification analysis. The results of the analysis for differentially expressed unigenes between the OA and NC groups are shown in Supplementary Figure S5f. Moreover, we performed enrichment analysis of differentially expressed unigenes between different groups, and produced the top 20 GO terms (Figure 5A). Finally, the most abundant metabolic pathway in the OA and NC groups was shown by KEGG analysis of differentially expressed unigenes (Figure 5B). Phosphoribosylaminoimidazole carboxylase activity, amino acid transmembrane transporter activity and extracellular region varied between the OA and NC groups (Figure 5A; Supplementary Table S8); and the two groups also differed in starch and sucrose metabolism, fatty acid biosynthesis, and glycerophospholipid metabolism (Figure 5B; Supplementary Table S8). In addition, we performed correlation analysis of the top 30 species by SparCC, and the results showed the correlation between the two dominant species with a network diagram (Supplementary Figure S5g).

## Discussion

An increasing number of studies have focused on the gut microbiome, and the cartilage-gut-microbiome axis has been proposed to be related to the pathogenesis of OA (Favazzo et al., 2020) for a few years. The relationship between OA and the gut microbiota by exploring a linkage between joint disorder and the metabolites in serum released by gut microbiota (Berthelot et al., 2019; Szychlinska et al., 2019; Favazzo et al., 2020) has been identified by several studies. Nevertheless, no thorough studies examining the variations in the gut flora between patients with knee OA and NCs have been published recently. Therefore, we identified alterations in the gut microbiome of knee OA and microbial diversity, and then provided new clues for the association between OA and the gut microbiota.

In previous studies, some intestinal bacteria were identified in human cartilage, for example, *Betaproteobacteria* were dominant in the cartilage from patients with OA, while *Actinobacteria* and *Clostridia* were found to be increased in the cartilage from normal controls (Dunn et al., 2018). The synovium and synovial fluid from patients with RA or OA also contained microbiota, such as *Bacteroides* (Zhao et al., 2018). In this study, the abundances of the species *Bacteroides uniformis*, *Bacteroides stercoris* and *Bacteroides vulgatus* were significantly decreased in knee OA. How did this microbiota get to the cartilage or synovium? It has been proposed that a translocation of the gut microbiota could occur in the deep areas of cartilage tissue, which could lead to OA cartilage injuries (Jeffries et al., 2016). However, how these microbiota can arrive at the cartilage, including the subchondral bone marrow, deep cartilage zone, chondrocytes and extracellular matrix, remains unknown. It could be that the metabolites produced by gut microbiota are toxic to chondrocytes and disturb the metabolism of cartilage (Jeffries et al., 2016). The primary cartilage loss in pathological changes of OA is mainly characterized by progressive degradation of the superficial zone of cartilage tissues, combined with severe subsequent inflammation. In addition, the osteochondral plate was the main site that vascularizes in the early stage of OA, which could cause the translocation of microbiota and their metabolic products to the deep cartilage tissue. The degradation and inflammation in cartilage from patients with OA could be induced by the germs in the deep layer cartilage due to the ability of the microbiota to act as pathogenic antigens, which can release the production of proinflammatory factors and transmitters in chondrocytes (Behera et al., 2008).

It is difficult to determine the linkage between a differential gut microbiota and OA or whether disorder is involved in OA through risk factors such as injury or metabolic diseases (Berenbaum, 2013; Courties et al., 2017). Currently, there are a large number of studies have focused on the important role of the gut microbiota in both patients with OA and OA animal models. The abundance of *Streptococcus species* was significantly positively related to increased pain in knee and joint injury severity, and the increased S*treptococcus species* abundance could lead to upregulation of bacterial products and metabolic products *in vivo* (Boer et al., 2019). In addition, *Streptococcus* has been found in reactive arthritis (Mcguire et al., 1977; Mackie and Keat, 2004; Barash, 2013; Tandon et al., 2013; Cunningham, 2014; Murillo et al., 2014). Several *Streptococcus* spp. were found to release the membrane vesicles (Brown et al., 2015), which can produce immunogenic products (Metcalfe et al., 2012; Bonnington and Kuehn, 2014) that can initiate macrophage activation by regulating TLR singling pathways, which has been proven to be related to the pain and joint inflammation of OA (Dahaghin et al., 2007; Metcalfe et al., 2012; Ohto et al., 2012). In our study, the abundance of *Streptococcus salivarius* was significantly increased in knee OA compared to NC. In a transplanted mouse study, Huang et al. found that the abundances of *Fusobacterium* and *Faecalibaterium* were increased and *Ruminococcaceae* was decreased in microbiota transplanted mice which were consistently correlated with the severity of OA and systemic biomarker concentrations (Huang et al., 2020). Guss et al. demonstrated that Toll-like receptor-5 deficient mice could develop metabolic syndrome because of changes in the gut microbiota (Guss et al., 2019). All above mentioned results confirmed the influence of gut microbiota and OA, and the open question remains as follows: How does this happen? A relationship between OA and metabolites or biomarkers, such as LPS released by gut microbiota has been found. Loef et al. found that there is a linkage between the concentration of fatty acids in serum and tissue injuries in knee and hand OA (Loef et al., 2020), and serum and synovial LPS levels have a positive relationship with joint destruction in knee OA (Huang et al., 2016).

## Conclusion

In conclusion, the results in this study showed that the genera, phyla and species changed in OA, which could present a comprehensive profile of the gut microbiota in patients with knee OA and offer evidence that the cartilage-gut-microbiome axis could play a crucial role in underlying the mechanisms and pathogenesis of OA.

## Data availability statement

The original contributions presented in the study are included in the article/Supplementary material, further inquiries can be directed to the corresponding author.

## Ethics statement

The studies involving human participants were reviewed and approved by the human ethics committee of Xi’an Jiaotong University. The patients/participants provided their written informed consent to participate in this study.

## Author contributions

XW, HZ, and XG concept and designed the study. MH, YN, YG, HZ, and RH collected sample. MH, YG, RH, SC, FZ, YL, FC, YW, SL, and CW conducted and collected data. YW, XW, and YN interpreted data and drafted manuscript. XG revised manuscript content. All authors contributed to the article and approved the submitted version.

## Funding

This study was financially supported by the National Natural Science Foundation of China (82273752 and 81620108026), Natural Science Basic Research Plan in Shaanxi Province of China (2023-JC-YB-704), the China Postdoctoral Foundation (2022 M712526 and 2021 M692543), and the Shaanxi Postdoctoral Foundation (2018BSHYDZZ47 and 2018BSHEDZZ96).

## Conflict of interest

The authors declare that the research was conducted in the absence of any commercial or financial relationships that could be construed as a potential conflict of interest.

## Publisher’s note

All claims expressed in this article are solely those of the authors and do not necessarily represent those of their affiliated organizations, or those of the publisher, the editors and the reviewers. Any product that may be evaluated in this article, or claim that may be made by its manufacturer, is not guaranteed or endorsed by the publisher.
