# Supplementary figures and images for: Altered gut microbiome profile in patients with knee osteoarthritis
Source: Front Microbiol. 2023 May 12;14:1153424. doi: 10.3389/fmicb.2023.1153424 (PMC10213253; doi:10.3389/fmicb.2023.1153424)

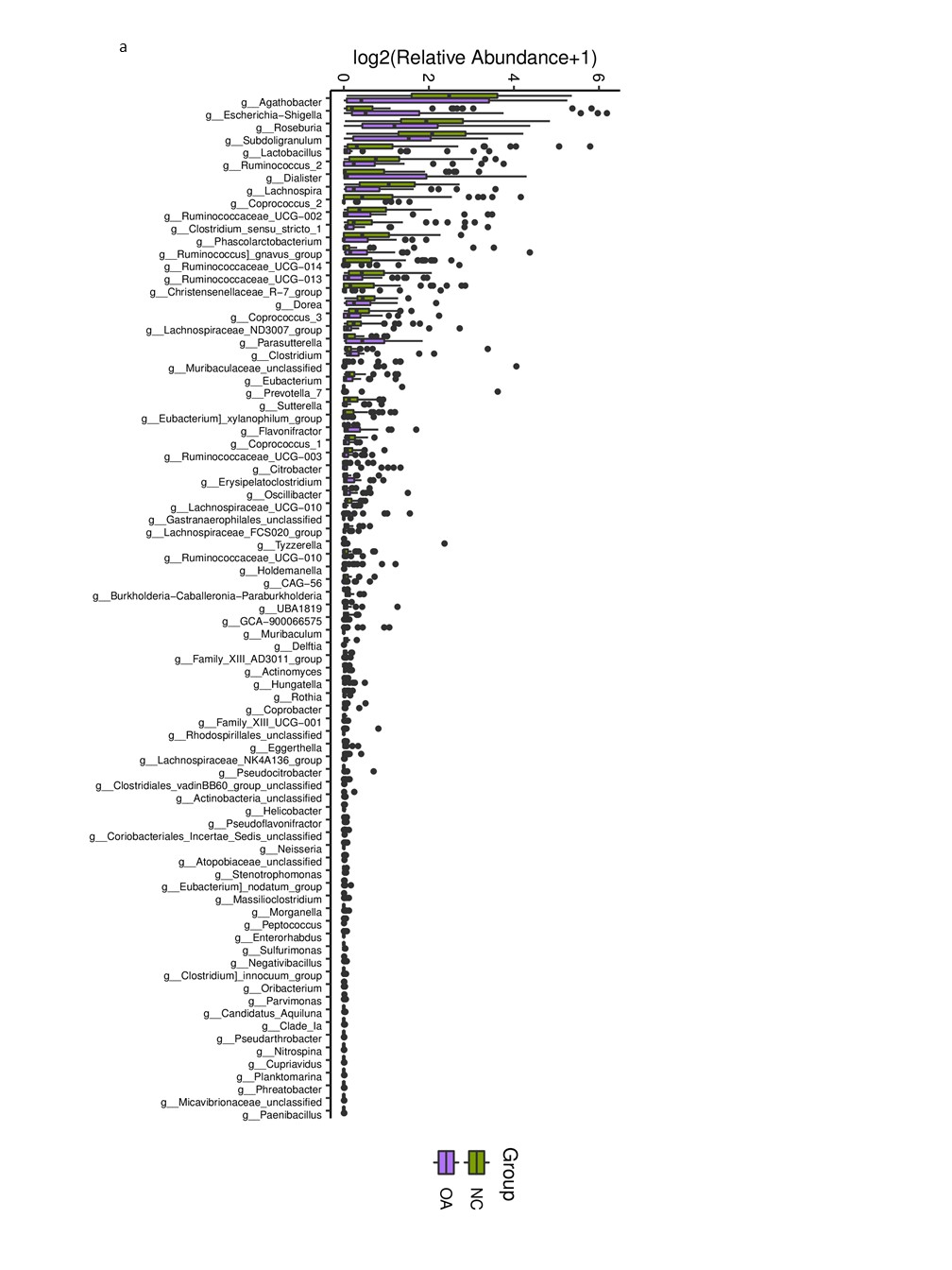

Supplement: Supplementary file 2 [file Image_1.JPEG]

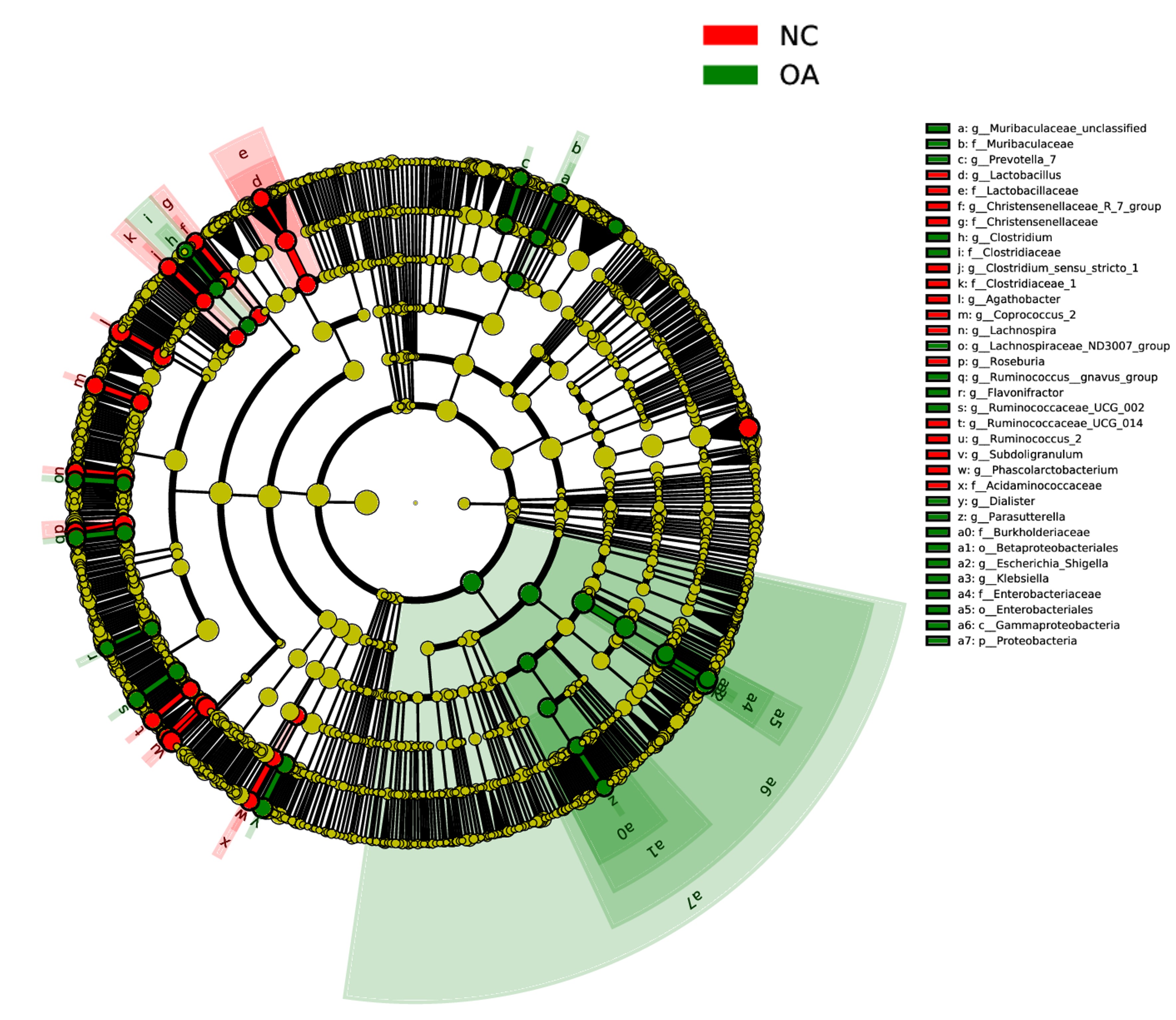

Supplement: Supplementary file 3 [file Image_2.JPEG]

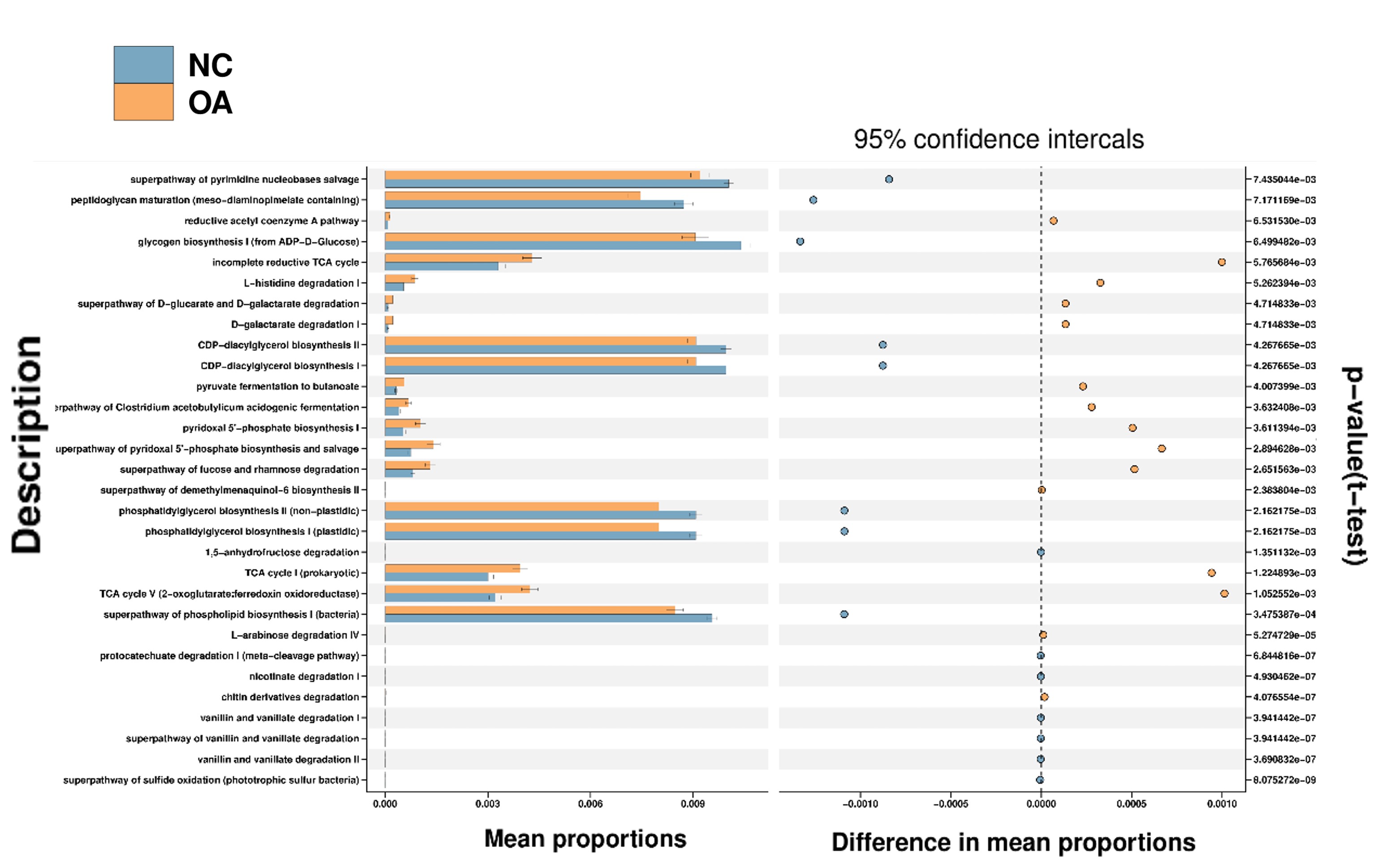

Supplement: Supplementary file 4 [file Image_3.JPEG]

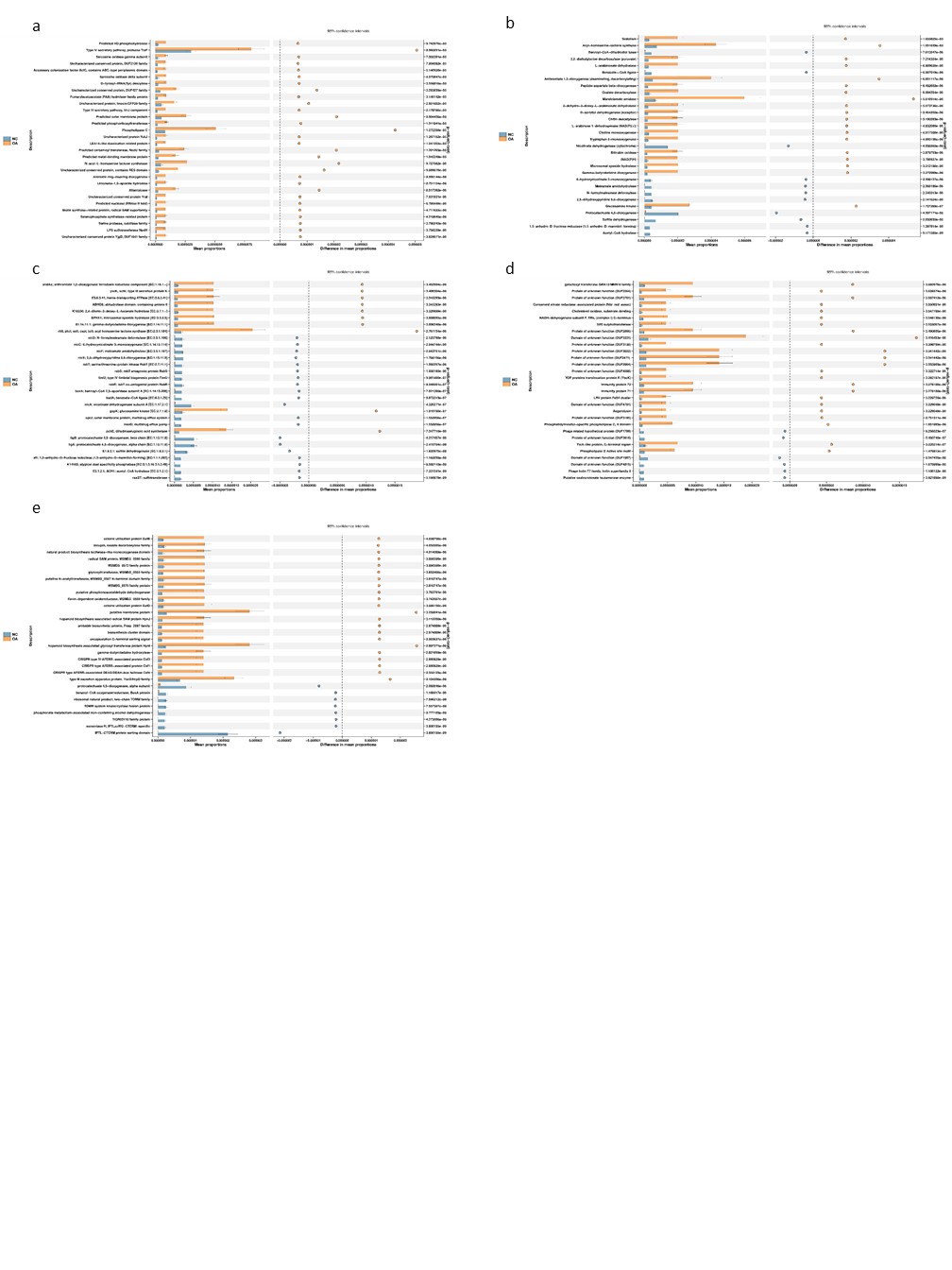

Supplement: Supplementary file 5 [file Image_4.JPEG]

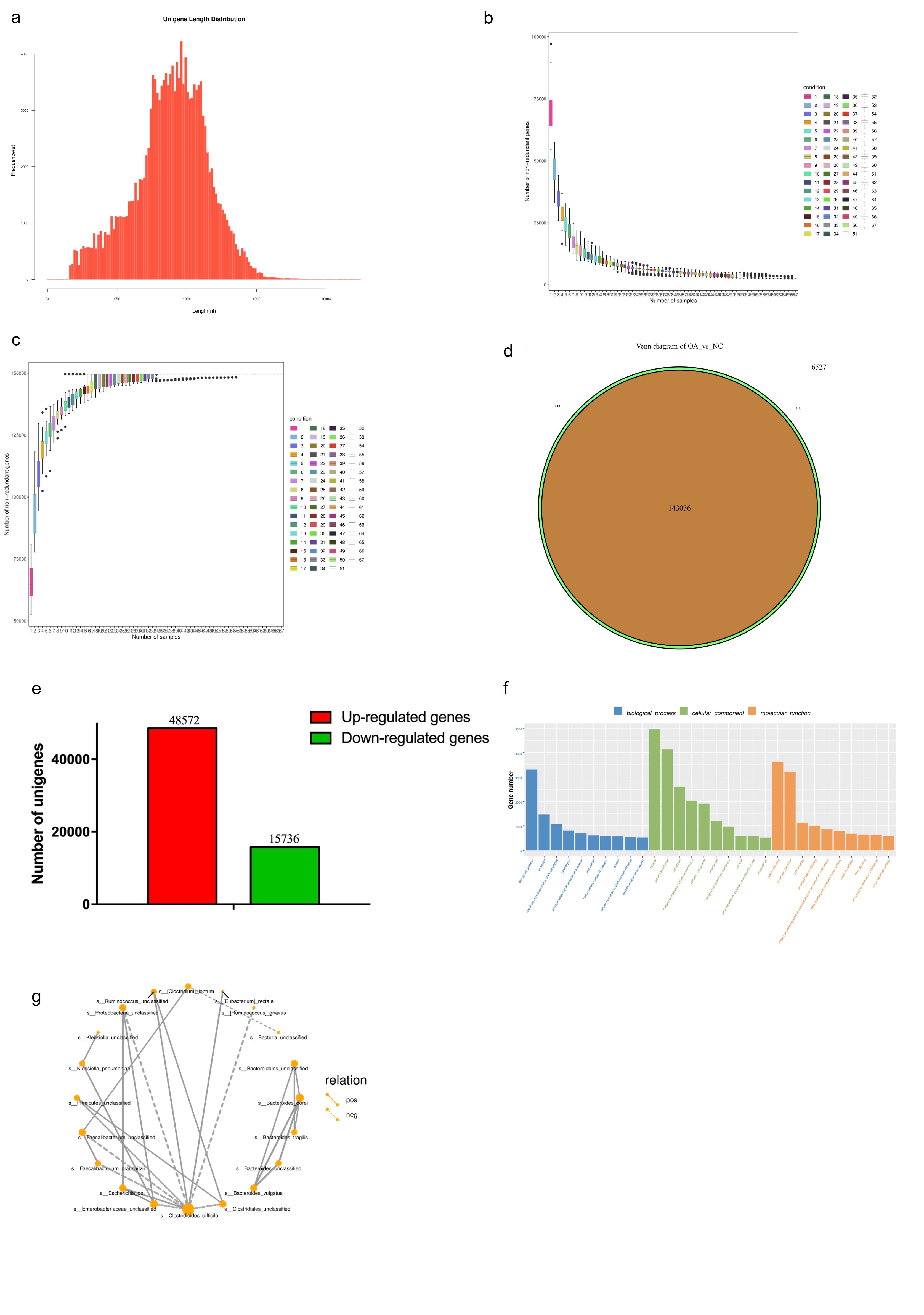

Supplement: Supplementary file 6 [file Image_5.JPEG]
